# Host and Aquatic Environment Shape the Amphibian Skin Microbiome but Effects on Downstream Resistance to the Pathogen *Batrachochytrium dendrobatidis* Are Variable

**DOI:** 10.3389/fmicb.2018.00487

**Published:** 2018-03-21

**Authors:** Andrea J. Jani, Cheryl J. Briggs

**Affiliations:** ^1^Department of Oceanography, University of Hawai’i at Mānoa, Honolulu, HI, United States; ^2^Department of Ecology, Evolution, and Marine Biology, University of California, Santa Barbara, Santa Barbara, CA, United States

**Keywords:** microbiome, community assembly, *Batrachochytrium dendrobatidis*, defensive symbiosis, chytridiomycosis, *Rana sierrae*

## Abstract

Symbiotic microbial communities play key roles in the health and development of their multicellular hosts. Understanding why microbial communities vary among different host species or individuals is an important step toward understanding the diversity and function of the microbiome. The amphibian skin microbiome may affect resistance to the fungal pathogen *Batrachochytrium dendrobatidis* (Bd). Still, the factors that determine the diversity and composition of the amphibian skin microbiome, and therefore may ultimately contribute to disease resistance, are not well understood. We conducted a two-phase experiment to first test how host and environment shape the amphibian skin microbiome, and then test if the microbiome affects or is affected by Bd infection. Most lab experiments testing assembly of the amphibian skin microbiome so far have compared sterile to non-sterile environments or heavily augmented to non-augmented frogs. A goal of this study was to evaluate, in an experimental setting, realistic potential drivers of microbiome assembly that would be relevant to patterns observed in nature. We tested effects of frog genetic background (2 source populations) and 6 natural lake water sources in shaping the microbiome of the frog *Rana sierrae*. Water in which frogs were housed affected the microbiome in a manner that partially mimicked patterns observed in natural populations. In particular, frogs housed in water from disease-resistant populations had greater bacterial richness than frogs housed in water from populations that died out due to Bd. However, in the experiment this difference in microbiomes did not lead to differences in host mortality or rates of pathogen load increase. Frog source population also affected the microbiome and, although none of the frogs in this study showed true resistance to infection, host source population had a small effect on the rate of pathogen load increase. This difference in infection trajectories could be due to the observed differences in the microbiome, but could also be due to other traits that differ between frogs from the two populations. In addition to examining effects of the microbiome on Bd, we tested the effect of Bd infection severity on the microbiome. Specifically, we studied a time series of the microbiome over the course of infection to test if the effects of Bd on the microbiome are dependent on Bd infection severity. Although limited to a small subset of frogs, time series analysis suggested that relative abundances of several bacterial phylotypes changed as Bd loads increased through time, indicating that Bd-induced disturbance of the *R. sierrae* microbiome is not a binary effect but instead is dependent on infection severity. We conclude that both host and aquatic environment help shape the *R. sierrae* skin microbiome, with links to small changes in disease resistance in some cases, but in this study the effect of Bd on the microbiome was greater than the effect of the microbiome on Bd. Assessment of the microbiome differences between more distantly related populations than those studied here is needed to fully understand the role of the microbiome in resistance to Bd.

## Introduction

Symbiotic bacterial communities are ubiquitous inhabitants of multicellular organisms and play important roles in the health and development of their hosts (Dethlefsen et al., 2007; Grice and Segre, 2012; Engel and Moran, 2013; Philippot et al., 2013). A great deal of research has focused on the microbiome of the human gut, but recent studies have begun to explore the microbiome of the skin, which in many animals is the largest organ in the body and a primary line of defense against pathogens (Abdallah et al., 2017). Shifts in the skin microbiome of humans are linked to psoriasis, atopic dermatitis, acne, and *Demodex*-associated rosacea, although it is unclear whether microbiome shifts are a cause or effect of these diseases (Grice, 2014). In a mouse model, skin-associated bacteria influenced skin-specific immune responses and severity of infection by the protozoan parasite *Leishmania major* (Naik et al., 2012). The links between microbiome composition and host health highlight the importance of understanding the factors that shape symbiotic communities. Understanding why bacterial communities differ can help identify the causes of imbalances (dysbioses) in the microbiome, and advance our ability to mediate disease susceptibility by facilitating the maintenance of healthy microbial communities.

In amphibians, understanding the assembly and dynamics of the skin-associated microbiome has implications for management of *Batrachochytrium dendrobatidis* (Bd), a fungal pathogen that infects the skin of amphibians and causes the potentially lethal disease chytridiomycosis. Bd has been implicated in amphibian declines worldwide (Berger et al., 1998; Lips et al., 2006; Briggs et al., 2010; Crawford et al., 2010; Kilpatrick et al., 2010; Vredenburg et al., 2010), and currently there are no practical methods to control the disease in wild populations. Recent research has raised the possibility that symbiotic bacteria present on amphibian skin might affect resistance to chytridiomycosis. Differences in the skin microflora of wild amphibians coincide with differences in apparent disease resistance (Woodhams et al., 2007, 2014; Rebollar et al., 2016; Jani et al., 2017). Probiotic treatments have been effective in some cases (Harris et al., 2009; Muletz et al., 2012; Kueneman et al., 2016), but not others (Becker et al., 2011, 2015; Woodhams et al., 2012; Kueneman et al., 2016). This variation in the efficacy of probiotic treatments is probably the product of multiple factors, a full discussion of which is beyond the scope of this article, but drivers of microbial community assembly are likely important. For example, factors shaping microbial communities may affect probiotics by determining the abundance of symbiotic microbes that suppress or facilitate the growth of the probiotic or alter its behavior. In addition, because probiotics are themselves microbes, their ability to colonize and persist is likely to be affected by the drivers shaping microbial community structure, stability and resilience. Better understanding of the factors controlling the amphibian skin microbiome, in both the presence and absence of Bd, are critical to refining microbially based approaches to mitigate the spread and impact of Bd.

A few recent studies have begun to examine the factors shaping the amphibian skin microbiome. Microbiomes differ between amphibian species, and this is at least partly host-controlled (McKenzie et al., 2012; Kueneman et al., 2014; Walke et al., 2014). Microbiome differences among populations of a given amphibian species (intraspecific differences) have also been observed (Kueneman et al., 2014; Walke et al., 2014; Belden et al., 2015; Costa et al., 2016; Rebollar et al., 2016; Jani et al., 2017), but the role of host and environment in driving those intraspecific differences is largely unknown. The skin of amphibians selects for a microbiome that is distinct from the environment (Jani and Briggs, 2014; Walke et al., 2014; Jani et al., 2017), but that is also influenced by environmental factors. Salamanders housed in sterile or non-sterile environments develop different microbiomes (Loudon et al., 2013; Knutie et al., 2017). The composition of aquatic environmental bacterial communities predicted the composition of the *R. sierrae* microbiome (Jani et al., 2017), and transplanting fire salamanders between aquatic habitats shifted their microbiomes (Bletz et al., 2016). We also know that Bd infection alters the microbiome (Jani and Briggs, 2014; Walke et al., 2015). In summary, we know that the microbiome differs among host species and is affected by dramatic changes in the environment (e.g., sterile versus non-sterile environment) or infection by Bd. However, we lack understanding of how microbiome variation that is specifically linked to host or environment subsequently influences resistance to disease.

The Sierra Nevada yellow-legged frog (*Rana sierrae*) is a species for which understanding microbiome assembly and interaction with Bd is particularly important. These frogs are severely threatened by Bd (Rachowicz et al., 2006; Vredenburg et al., 2007, 2010). However, *R. sierrae* populations exhibit variation in their response to Bd infection: many populations have rapidly been driven extinct by the pathogen (“die-off populations”), but some populations appear to resist large-scale lethal disease (“persistent populations”) (Briggs et al., 2010; Vredenburg et al., 2010; Knapp et al., 2011, 2016; Jani et al., 2017). Persistent and die-off populations harbor different bacterial communities (Woodhams et al., 2007; Jani et al., 2017). Understanding the drivers of microbiome variation and microbiome – Bd interactions in *R. sierrae* may inform efforts to conserve the species. Here, we present a study that experimentally tested the roles of host and environment in shaping the *R. sierrae* microbiome in the presence and absence of Bd. We then link host and environmental effects on the microbiome with subsequent response to Bd exposure. We address four overarching questions about the bacterial component of the microbiome (hereafter simply “microbiome”): (1) Do innate (e.g., genetic) differences in the host shape intraspecific variation in the microbiome? (2) Does the aquatic habitat shape intraspecific variation in the microbiome? (3) Does the microbiome variation that results from environmental and host differences affect downstream resistance to Bd? (4) What is the evidence for the microbiome altering Bd infection dynamics, as opposed to Bd altering the microbiome?

## Materials and Methods

### Collection of *R. sierrae* and Lake Water

All equipment, including nets and shoes, that was likely to come into contact with frogs or lake water was disinfected (incubated with 0.1% quaternary ammonium solution for at least 5 min) before beginning work in any field site. Small or sensitive equipment was disinfected with 70% ethanol. *R. sierrae* were from existing laboratory colonies, originally collected as eggs or tadpoles from two populations in the Sierra Nevada. All *R. sierrae* collections were made during the 2010 field season under research permits from the National Park Service and United States Forest Service, and subsequently housed at the University of California, Santa Barbara (UCSB) in facilities certified by the UCSB Institutional Animal Care and Use Committee (IACUC). United States Fish and Wildlife permits were not applicable at the time of this study, which was prior to the 2014 listing of *R. sierrae* as endangered by the United States Fish and Wildlife Service. One population was located in a lake of Humphrey’s Basin (Sierra National Forest, collected as tadpoles), and the other population was located in Dusy Basin (Kings Canyon National Park, collected as eggs). Both populations have experienced Bd-induced die-offs to the point of population extirpation. We targeted “stranded” eggs for collection, meaning that the eggs were found in microhabitats where they were unlikely to survive to metamorphosis due to high risk of desiccation or predation. At the time of this experiment, the frogs from Humphreys Basin were larger than those from Dusy Basin (*P* < 0.0001), and we cannot definitively distinguish effects of genetic background from possible effects of frog age or size. We chose to work with these existing colonies despite the age difference rather than collect new individuals of a threatened species from the wild. However, we do not expect the age difference to have measurable effects because previous work found no difference in the microbiomes of juvenile and adult *R. sierrae* and *Rana cascadae* (Jani and Briggs, 2014; Kueneman et al., 2014). Furthermore, in the current study the age difference was small, and all individuals were juveniles (post-metamorphosis, but pre-reproductive).

Lake water to be used as aquatic habitat treatments in the experiment was collected from six Sierra Nevada lakes. Three of the water collection sites were inhabited by persisting *R. sierrae* populations. The other three sites had been previously inhabited by *R. sierrae* populations that had declined due to Bd one to 3 years prior. We refer to these as “persistent” and “die-off” populations, respectively. Water was collected in 2.5-gallon (∼10 l) collapsible polyethylene jugs (Cubitainer), which were washed with 0.1% quaternary ammonium solution 128, triple-rinsed with tap water, soaked in tap water for at least 15 min, then rinsed again. Jugs were additionally triple-rinsed in lake water at the collection site before being used for lake water collection. Because most *R. sierrae* populations are located in back-country areas far from roads, water was transported from field sites on foot. Lake water was stored at 4°C at the Sierra Nevada Aquatic Research Laboratory for up to 8 days, then transported by automobile, with water jugs held in insulated containers with ice, to UCSB where it was stored at 4°C until use. The experiment was initiated 3 days after arrival at UCSB. Prior to being added to experimental frog tanks, all lake water was filtered (1.2 μm pore size) to remove any Bd cells that may naturally occur in lake water while retaining bacteria in the lake water. Bd zoospores are 3–5 μm in diameter and sporangia are much larger. Based on published size distributions of freshwater bacteria (Šimek and Chrzanowski, 1992), we estimate that at least 80% of planktonic bacteria in our lake water should pass through 1.2 micron pores and remain in the water after filtration.

### Preparation of Bd and Sham Inocula

Four Bd strains (TST75, CBJ4, CJB5, CJB7) isolated from *R. muscosa* or *R. sierrae* (which were formerly classified as the same species) were used in this experiment. A mixed-strain inoculum was used to minimize any strain-specific biases in infection dynamics. Bd cultures from cryo-archived stocks were grown in 1% (w/v) tryptone liquid medium. Once viability was confirmed, cultures were passaged to agarose tryptone media in petri plates (10 g L^-1^ tryptone, 10 g L^-1^ agar). Tryptone plates without Bd added were prepared in parallel with cultures as a sham inoculum. Cultures and sham plates were harvested after 4–6 days of growth by flooding plates with sterile water for 45 min to induce release of zoospores from sporangia and then collecting the zoospore suspension. To avoid introducing Bd culture medium to frog tanks along with Bd inoculum, zoospores and sham inocula were rinsed three times by gently pelleting (500 G, 5 min) in 50 ml conical tubes, drawing off the supernatant, and resuspending cells (or sham inoculum) in 35 ml sterile water. This is important because culture medium could affect microbial communities in the experiment. After rinsing, cells were counted visually at 200X magnification on a compound light microscope using a hemocytometer. The four strains were pooled, with an equal concentration of each strain, and the cell suspension was diluted to 200,000 cells mL^-1^ and used immediately.

### Experimental Design

We conducted a two-phase experiment to test determinants of microbiome composition and downstream effects on resistance to Bd infection. The experiment was conducted September – December 2011. A previous publication (Jani and Briggs, 2014) included data from the second phase of this experiment to test if Bd infection (as a binary variable) alters the microbiome, to test effects of captivity on the microbiome, and to compare dynamics in the laboratory and field. The former study did not analyze variation among infected frogs in the experiment, analyze time series data, test if the microbiome predicts Bd infection, or analyze any of the community assembly data from Phase I of the experiment. An outline of the experiment timeline is provided in Supplementary Figure S1. *Phase 1 – microbiome assembly:* The first phase of the experiment tested effects of aquatic environments and host genetic background on the *R. sierrae* skin microbiome. To vary host population, forty-two frogs from each of the two *R. sierrae* populations were included in the study. To vary the aquatic habitat, each frog was housed in an individual tank with water from one of seven Water Sources, which included natural water collected from six different lakes and one sterile water treatment. We chose lake water as our focus because *R. sierrae* are aquatic amphibians most often found either in lake water or basking on adjacent rocks. The high elevation Sierra Nevada lakes where the species is found are rocky, oligotrophic habitats with relatively little soil or vegetation, so lake water is a dominant feature of these frogs’ environment. Furthermore, we previously showed that the bacterial communities present in lake water are correlated with the bacterial communities found on *R. sierrae* (Jani et al., 2017). In addition to the six lake water treatments, one sterile water treatment was prepared by autoclaving bottled drinking water (Arrowhead); sterility was confirmed by plating cooled 100 μL aliquots onto R2A and LB agar plates. We use the term “Water Sterility” to refer to whether water was living lake water or sterile bottled water. Lake water was collected from six Sierra Nevada lakes, of which three were inhabited by persistent *R. sierrae* and three were once inhabited by die-off populations. We use the term “Lake Water Type” to refer to the type of population site from which lake water was collected: “persistent” or “die-off” Lake Water Type indicates water collected from the geographic site of a persistent or die-off population, respectively. Note that “Lake Water Type” and “Water Source” (described above) are distinct variables: there are 7 Water Sources; six of them are from lake water, and these six are evenly divided among persistent and die-off Lake Water Types. We used a fully crossed design with 14 treatments (2 Frog Sources × 7 Water Sources), and six replicate frogs assigned to each treatment. For 2 weeks prior to beginning the experiment, animals from both populations were co-housed in three large common-garden tanks to standardize any pre-experiment environmental effects (Jani and Briggs, 2014). Non-sterile, bottled drinking water was used to provide an aquatic habitat in the pre-experiment common-garden tanks. Immediately before beginning the experiment, each frog was treated with 3% hydrogen peroxide (50 ml in a 100 ml container) for 30 s, and then rinsed thoroughly with sterile water (two 100-ml sterile water baths lasting 2 and 8 min, respectively), in an attempt to reduce and standardize the bacterial community present on the skin of frogs (e.g., Harris et al., 2009). (Hydrogen peroxide baths have been used in previous laboratory studies of the amphibian skin microbiome, however, in the current study we found that the treatment caused stress for *R. sierrae*. We therefore advise caution in the use of this method.) To initiate the experiment, twelve frogs (six from each population) were randomly assigned to each of the seven Water Sources. All frogs were housed in individual tanks, each containing 250 ml of water from one of the seven Water Sources, for the duration of the experiment. Tanks were positioned at a slant so that the tank floor was partially submerged in water, offering aquatic and basking space. Each frog was offered 7 crickets once per week, and tank water was changed after feeding to minimize the establishment of food-associated bacteria in experimental tanks. All tank water changes were done using the assigned experimental water source for each frog. Tanks were randomly assigned positions in environmental chambers maintained at 17°C with a 12 h photoperiod. Phase 1 was allowed to run for 3 weeks, based on our assumption that this would be ample time for effects of the water treatments on the microbiome to be detectable. *Phase 2 – Bd challenge:* Three weeks (21 days) after beginning the experiment, 42 of the frogs (3 frogs from each Frog Source × Water Source treatment) were challenged with Bd (200,000 zoospores per frog for three consecutive days). The dose of 200,000 zoospores is similar to previous studies (e.g., Stice and Briggs, 2010). The remaining 42 frogs served as Bd-free controls and received a sham inoculum. Frogs were monitored daily throughout the experiment. At 60 days post-exposure (82 days after initiating the experiment), the experiment was concluded and surviving frogs were cleared of Bd infection by treatment with Itraconazole (1.5 mg/L bath, 7 min daily for 11 days). Infection status for all frogs was confirmed by quantitative PCR analysis of skin swabs (described below in “Quantifying Bd Load”).

To minimize the use of *R. sierrae* for experiments while maximizing information gained, we designed this experiment to address multiple research questions: (1) How do host and environment affect the microbiome? This question is addressed in the first 3-week phase of the experiment. (2) How does variation in the skin microbiome affect resistance to Bd? This question is addressed by examining how variation in the microbiome present at the end of phase 1 (just prior to Bd challenge) is correlated with downstream severity of Bd infection. (3) Does the severity of Bd infection drive changes in the microbiome? To address this question, we test if the *R. sierrae* microbiome changes as Bd infection severity increases through time. (4) Does Bd infection *per se* (infected versus uninfected) alter the microbiome? This question is addressed by comparing the microbiomes of Bd-infected and Bd-free (control) frogs after Bd challenge. The first three research questions are the focus of the current article, while the fourth question is addressed elsewhere (Jani and Briggs, 2014).

### Data Collection

Microbes present on frog skin (including bacteria and Bd) were sampled from all frogs immediately prior to placing them in experimental tanks (at the initiation of the experiment), and at least once weekly thereafter. In addition, frogs were swabbed twice weekly during Phase 1, before Bd challenge. A subset of frogs was also swabbed twice prior to beginning the experiment: immediately before (subset of frogs) and after (all frogs) hydrogen peroxide treatment. New nitrile gloves were worn for each frog handled, and frogs were rinsed twice with 60 ml sterile water before swabbing the skin using a sterile synthetic swab (Medical Wire and Equipment Company) following standard protocols (Briggs et al., 2010; Jani and Briggs, 2014). Swab buds were immediately placed in sterile microcentrifuge tubes on ice, and then frozen within 1 h of collection. We monitored symptoms of chytridiomycosis, including weight loss, inappetence, and excessive shedding of skin. We recorded snout-to-vent length and weight of all frogs before infection and at 6 and 8 weeks post-exposure. We counted the number of crickets eaten by each frog weekly and scored the amount of shed skin present in tank water using a qualitative 3-level rating system: no shed skin, moderate amount, or copious amount of shed skin observed in tank water. Prior to adding lake water to frog tanks, bacteria present in lake water were sampled by filtering 250 ml from each lake Water Source through a 0.22 μm pore polyethersulfone filter (Sterivex-GP; Millipore). Filters with samples of aquatic bacteria were frozen immediately.

### DNA Extraction

Total DNA (including bacterial and Bd DNA) from frog skin swabs was prepared for PCR using Prepman Ultra as described previously (Jani and Briggs, 2014). Prepman Ultra minimizes the amount of DNA lost in the extraction process and has been used effectively in other studies (Jani and Briggs, 2014; Jani et al., 2017). DNA from aquatic bacterial samples was extracted following Nelson (2009). Tubes with extraction reagents, but without sample, were included as extraction negative controls.

### Quantifying Bd Load

We quantified Bd loads from all swabs collected at the beginning of the experiment, immediately prior to Bd challenge, and weekly thereafter until the end of the experiment. Bd load (also referred to as Bd infection intensity) was measured by quantitative PCR (qPCR) applied to skin swab DNA samples as described previously (Boyle et al., 2004), with Bd standards provided by the laboratory of Alex Hyatt (CSIRO, Australia). Bd load represents the number of Bd cells in a swab sample, and when combined with a standardized swabbing method represents the total pathogen load of each frog. No-template controls and extraction negative controls were included in all PCR runs. All frogs in the Bd- treatment remained free of Bd, with the exception of one frog, which became contaminated with Bd and was excluded from analyses.

### Selection of Samples for Bacterial 16S Sequencing

Due to the labor requirements and cost of sequencing, it was not feasible to analyze bacterial communities from all samples collected in the experiment (84 animals sampled weekly for up to 11 weeks). Instead, we analyzed bacterial communities across all frogs at three time points: (1) Immediately before beginning the experiment, to confirm that no differences exist between experimental water treatments before beginning the experiment. This is referred to as day 0 of the experiment. (2) After 21 days of inhabiting the various water treatments, but immediately before Bd challenge. This time point is referred to as day 21 of the experiment or 0 days post-exposure (PE). (3) After Bd infection (day 43 of the experiment, or 21 days PE). Samples of aquatic bacteria in stored lake water were also analyzed on three dates roughly corresponding to time periods when frog skin bacteria were analyzed. The 21 days PE time point was chosen for two reasons. First, Bd loads at 3 weeks PE were representative of loads observed during epidemics in the wild (Jani and Briggs, 2014; Jani et al., 2017). Second, mortality began between the sampling dates at 3 weeks and 4 weeks PE, so 3 weeks PE is the latest sampling point at which analyses are not biased by missing individuals. To analyze progressive effects of Bd loads as they increased through time while minimizing variation due to Water Source and Frog Source, we also sequenced weekly samples of 6 frogs: 3 Bd-infected and 3 unexposed animals, all from the Marmot Frog Source and with the same Water Source.

### Bacterial Community Sequencing and Bioinformatic Processing

The bacterial communities present on frog skin and in lake water were characterized by sequencing a portion of the 16S gene, as described in detail in Jani and Briggs (2014). Briefly, the V1–V2 region of the 16S gene was amplified using barcoded primers with sequencing adapters, and PCR products were purified, pooled in equimolar quantities, and sequenced on a Roche/454 GS FLX instrument using Titanium chemistry. Negative controls and no-template controls were included. The program mothur v 1.30 (Schloss et al., 2009) was used to quality-filter sequences, align them to a non-redundant representative subset of the SILVA v111 SSU Ref 16S curated alignment database (Nelson et al., 2014), and cluster sequences into 95% sequence identity operational taxonomic units (OTUs) and phylotypes at the most specific classification level available, which is generally the “genus” level. We grouped OTUs at the 95% sequence identity level because 95% identity across the sequenced V1–V2 region of the 16S rRNA gene best approximates 97% identity across the entire 16S gene, a standard benchmark for assigning bacterial taxa (Schloss, 2010). Phylotype refers to a grouping of sequences based on taxonomic classification rather than percent identity.

Sequences were classified using the Bayesian classifier of Wang et al. (2007) and each OTU was assigned a consensus taxonomy from SILVA v111 (Pruesse et al., 2007; Quast et al., 2013). Weighted Unifrac distance (Lozupone and Knight, 2005) was calculated from OTU relative abundance data to quantify the degree of phylogenetic difference among bacterial communities from different samples. To estimate bacterial community richness and diversity, the number of observed OTUs (S_OBS_), Chao’s richness estimate (Chao, 1984), Shannon diversity, and Shannon evenness were calculated after subsampling to 500 sequences per sample. Unifrac distances and community diversity were calculated based on 95% identity OTUs. To identify specific bacterial taxa that were correlated with variables of interest (Frog Source, Water Sterility, Lake Water Type, or Bd infection or load) we conducted tests on bacterial phylotypes rather than 95% identity OTUs because we have found that comparisons of specific bacterial groups among studies is better facilitated by phylotype classification (e.g., genus) than percent identity OTU. We note that results from phylotype and 95% identity OTU analyses were consistent (Supplementary Table S1). DNA sequence data have been deposited in the National Center for Biotechnology Information Sequence Read Archive (NCBI SRA, accession number SRR1598944).

### Testing Effects of Frog Source and Aquatic Environment on the Microbiome

We tested for effects of Water Source and Frog Source on the *R. sierrae* microbiome in the absence of Bd by examining bacterial communities from frogs sampled after 21 days in the water source treatments (before Bd exposure). Our response variable was Unifrac distance, a measure of phylogenetic dissimilarity between bacterial communities. We tested the effects of Frog Source (Dusy Basin or Humphreys Basin) and Water Source (7 sources) as fixed factors using permutational multivariate ANOVA (PERMANOVA). The Frog × Water interaction was not significant and was dropped from the model.

In addition to testing effects of the seven Water Sources, we tested two specific hypotheses about how the aquatic environment might affect the *R. sierrae* microbiome. Based on field observations (Jani et al., 2017) we hypothesized that the aquatic habitat helps shape differences in the microbiome between *R. sierrae* populations that persist or die out due to Bd. We predicted that frogs housed in water from lakes inhabited by persistent populations would harbor different bacterial communities than frogs housed in water from lakes where die-offs occurred. We used PERMANOVA with Frog Source and Lake Water Type (water from a persistent or die-off population) as main effects and Water Source nested in Lake Water Type to account for the fact that each class of water (persistent or die-off) encompasses three different Water Sources. We also tested if the frog skin microbiome differed between frogs housed in sterile water and frogs housed in non-sterile lake water. For this test we used PERMANOVA with Frog Source and Water Sterility (“sterile” or “live”) as main effects and Water Source nested in Water Sterility to account for the fact that the live water treatment encompasses six distinct Water Sources. Analogous parametric (ANOVA) models were used to test effects of water treatments on bacterial diversity, using the four diversity metrics described in the previous section (“*Bacterial Community Sequencing and Bioinformatic Processing”*).

We repeated tests of effects of Frog Source, Water Sterility, and Lake Water Type on the microbiome using data collected on day 43 of the experiment (21 days PE). The objective here was to test if Bd disturbance overrides the effects of host and aquatic environment in shaping the microbiome. For analyses of data after Bd exposure, Bd infection status (Bd+, Bd-) was included in ANOVA and PERMANOVA models to account for the effect of Bd on microbiome diversity and composition, respectively.

To confirm that no effect of water treatments existed before the water treatments were applied, the effects of Water Source and Frog Source on the microbiome were also tested using samples collected at the beginning of the experiment (21 days pre-infection, immediately before placing frogs in their respective experimental tanks with lake water).

### Testing Effects of Frog Source and Water Source on Bd Infection Severity

Bd load data collected from swabs before Bd challenge and weekly thereafter were used to examine infection trajectories through time. Only Bd load data from the 42 frogs in the Bd+ treatment (not the Bd- treatment) were analyzed for Bd infection trajectories because we were interested in effects of experimental treatments on variation in Bd trajectories given that frogs were exposed to Bd. We used repeated measures ANOVA (RM-ANOVA) to test for differences among experimental treatments in the rate of increase of Bd load through time. Data for RM-ANOVA were restricted to swabs collected between 0 and 21 days PE because 21 days PE was the latest time point for which all frogs were still alive. As with tests for treatment effects on the microbiome described above, we tested effects of Frog Source and Water Source and then followed up with specific hypothesis tests for effects of Water Sterility and Lake Water Type.

### Testing Direct Correlations Between Bacterial Communities and Bd Infection Severity

An objective of this study was to tease apart the effects of Bd on the microbiome from effects of the microbiome on Bd infection. For this analysis we included only frogs in the Bd+ treatment, since we are interested in how the microbiome affects the severity of infection given that a frog is exposed to the pathogen. We used Mantel tests to calculate Spearman rank correlation coefficients between the distance matrices based on the microbiome and the distance matrix based on differences in Bd load (3 weeks PE). The Mantel test quantifies the correlation between two distance matrices using permutation methods, therefore Bd load data were converted to pairwise (among-sample) Euclidean distances for Mantel tests. Bacterial community composition is already in the form of the Unifrac distance matrix. To test if the microbiome affects disease resistance, we tested for a correlation between the bacterial distance matrix prior to infection (at 0 days PE) and the Bd distance matrix 3 weeks after exposure (21 days PE). To test if Bd infection severity affects bacterial communities, we tested for a correlation between the Bd load matrix and the bacterial distance matrix, both measured at 21 days PE. The strength of the two effects (Bd effect on microbiome and vice versa) was examined by comparing *p*-values and correlation coefficients of the two Mantel tests. We used parametric tests to determine univariate correlations between diversity metrics and Bd load.

### Identifying Bacterial Phylotypes Associated With Host, Environment, or Bd Infection

The above analyses test for whole-community patterns in the microbiome. We also identified individual bacterial phylotypes that are associated with frog source or water treatments using parametric ANOVA with structure analogous to the corresponding PERMANOVA models described above, as follows: To test effects of Frog Source and Lake Water Type in the absence of Bd (using data from 0 days PE), we used ANOVA with Frog Source, Lake Water Type, and Water Source (nested in Lake Water Type) as the predictors, and phylotype relative abundance as the response. To test the effect of Water Sterility, we included Water Sterility, Water Source (nested in Water Sterility), and Frog Source as predictors and phylotype relative abundance as the response. To test the effect of Bd load (continuous variable) on phylotype relative abundance, using swabs from day 21 PE and only from frogs that were exposed to Bd, we included Frog Source and Water Source as random variables, Bd load as the predictor and phylotype relative abundance as the response. To test effects of initial abundance of each phylotype on subsequent Bd infection severity, using only data from frogs exposed to Bd, we included Frog Source and Water Source as random variables, phylotype relative abundance on day 0 PE as the predictor, and Bd load on day 21 PE as the response.

### Time Series Data to Test Effect of Bd Load on Bacteria

We further tested for effects of Bd on the microbiome by analyzing weekly samples through time, while eliminating variation due to Frog Source and Water Source. To do this, we selected one Frog Source × Water Source group, consisting of the 6 frogs from the Marmot population that were housed in water from a single lake. Three of the frogs were Bd-exposed and three were unexposed. The choice of which Water Source × Frog Source treatment to sequence was arbitrary because, prior to the 16S sequencing run, we had no data to suggest that any particular lakewater-frog treatment would be more informative than others. However we did avoid the sterile water treatments because those were expected to be less representative of wild frogs than the lake water treatments. We sequenced weekly samples from the 6 frogs from 0 to 28 days PE. (In this subset of frogs, mortality began between 4 and 5 weeks PE, hence this analysis extends to 28 days PE rather than the 21 day PE time point used when analyzing all frogs together). This complete time series analysis was not performed for the whole set of 84 frogs due to the cost of sequencing so many samples. By analyzing frogs from the same frog source and water source, we reduced variation due to frog and water source, allowing a clearer analysis of variation due to Bd infection. We tested for change over time individually for each phylotype that was represented by at least 10 reads across the 6 frogs. We first conducted repeated measures ANOVA to test for an interaction between Bd infection and time (Bd^∗^time effect). However, all tests resulted in *P* > 0.05 for the Bd^∗^time effect, possibly because of the low sample size relative to the number of time points (3 frogs per treatment, 5 time points). Therefore, as a rough test to identify phylotypes that are likely affected by Bd, we also conducted a simple correlation between time (day) and relative abundance of each phylotype. This simple correlation does not account for non-independence of an individual frog on different days as repeated measures ANOVA does, and we present the results as suggestive trends.

### Statistical Details

Parametric statistical analyses (ANOVA, repeated measures ANOVA) were performed in JMP (SAS Institute Inc., Cary, NC, United States, 1989–2007). Non-metric multidimensional scaling (NMDS) ordination and permutation-based analyses of bacterial community composition, including PERMANOVA and permutational Mantel tests, were conducted in Primer-e v6 (Clarke and Gorley, 2006). Bd load data were log_10_(X+1) transformed for all analyses. For analyses of abundances of individual phylotypes, relative abundance data were arcsine(square root) transformed, and only phylotypes represented by at least 10 reads in the sequencing run were analyzed (177 phylotypes). For time series analysis, we included only phylotypes with at least 10 reads across the 6 frogs for which we had time series microbiome data (95 phylotypes). Significance of analyses of relative abundances of individual phylotypes was determined after accounting for the false discovery rate (FDR) using the program *Q*-value (Storey, 2002), with a threshold of *P* < 0.05 and *Q* < 0.05 (Storey and Tibshirani, 2003). In some cases we discuss results with *P* < 0.05 and 0.05 ≤ Q < 0.1, which we annotate as marginally significant.

### Pooling of Data for Graphical Display

We use NMDS ordination to visualize multidimensional data (e.g., Unifrac distances). In NMDS, the stress associated with an ordination provides a measure of the distortion of the data incurred when multidimensional data are represented in fewer (usually 2) dimensions. In this study, NMDS stress for most ordinations was high (up to 0.22). This may be due to very high variability in the data. To reduce stress in the ordinations, we pooled data across the three replicate frogs within each treatment and day. Ordination plots therefore display data pooled within treatment and day, while all statistical analyses were conducted on the unpooled data, as specified in descriptions of the statistical models. Ordinations are for data visualization only and do not affect statistical results.

## Results

### Frog Source and Water Source Contributed to Microbiome Composition

**FIGURE 1:**
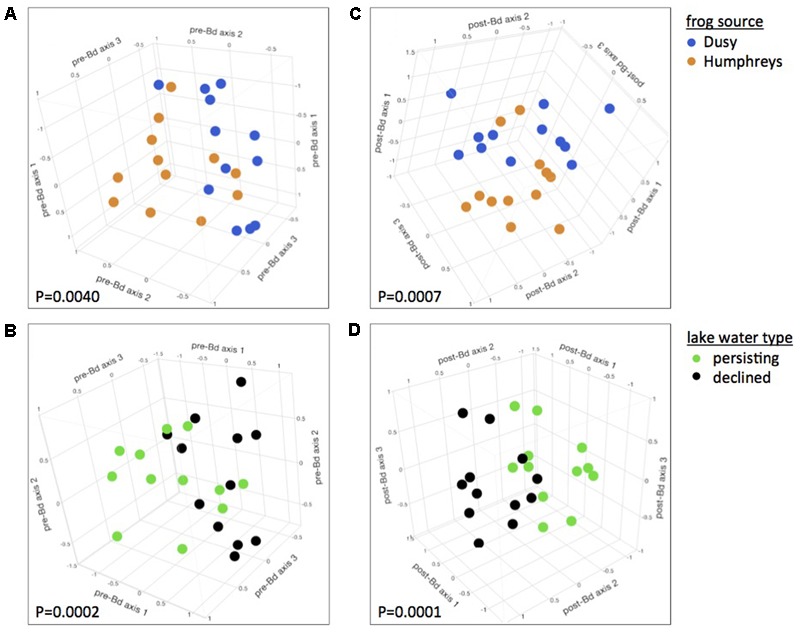
Effects of host and aquatic environment on the skin microbiome. Skin bacterial communities were significantly different between frogs from the two source populations prior to Bd infection **(A)** and differences persisted after Bd infection **(C)**. Frogs housed in water from persistent and die-off field sites had different microbiomes, **(B)** and these differences persisted after Bd infection **(D)**. Plots are 3-dimensional NMDS ordinations of *R. sierrae* skin-associated bacterial communities, pooled by treatment, sampled on day 21 of the experiment (after 3 weeks of water treatments, and before Bd infection, left panels), and day 43 (21 days after Bd exposure, right panels). Plots are rotated to display treatment differences. Marker colors indicate Frog Source (top) or Lake Water Type (bottom). NMDS stress: **(A,B)** 0.06; **(C,D)** 0.1.

At 0 days PE (before Bd challenge but 21 days after initiation of Water Source and Frog Source experimental treatments), microbiome composition was significantly affected by both Water Source and Frog Source. Frogs from the two source populations harbored significantly different bacterial communities, and this was true regardless whether all treatments or only lake water treatments were considered (PERMANOVA for all treatments: Pseudo-*F*_1,75_= 2.92, *P* = 0.0166, and for only lake water treatments: Pseudo-*F*_1,65_= 4.03, *P* = 0.0040, **Figure 1A**). *R. sierrae* microbiome composition differed based on Lake Water Type (population persistence or die-off at the field site from which water was collected, Pseudo-*F*_1,65_= 7.18, *P* = 0.0002, **Figure 1B**). In analyses focused on water sterility, frogs housed in sterile water harbored different bacterial communities than frogs housed in live water (Pseudo-*F*_1,75_= 4.52, *P* = 0.0009, Supplementary Figure S2).

Microbiome composition of samples collected at the start of the experiment (immediately before placing frogs in their respective water treatments) showed no effect of Water Source (*P* > 0.05), confirming that the effects of Water Source observed after application of experimental treatments was indeed due to the treatments. In contrast, the composition of microbial communities did differ between the two Frog Sources at the start of the experiment (Pseudo-*F*_1,75_ = 5.30, *P* = 0.0035), indicating that effects of Frog Source on the microbiome are at least partially robust to normalizing forces such as shared aquatic environments created by the common-garden pre-experiment tanks or the pre-experiment hydrogen peroxide treatments employed in this study. When analyzing each phylotype individually, we identified a number of phylotypes for which relative abundance was associated with Frog Source, Lake Water Type, or Water Sterility (Supplementary Table S2).

Three weeks after Bd infection, skin bacterial communities still differed based on Frog Source and Water Source. Bacterial community composition differed between frogs from the two source populations (Pseudo-*F*_1,64_ = 4.38, *P* = 0.0007, **Figure 1C**) as well as between frogs housed in water from field sites with different disease dynamics, i.e., population persistence or die-off (Pseudo-*F*_1,64_ = 7.95, *P* = 0.0001, **Figure 1D**). Microbiomes also differed between sterile and live water treatments (Pseudo-*F*_1,74_ = 3.92, *P* = 0.0007, Supplementary Figure S2).

### Bd Infection Trajectories and Frog Survival Were Subtly Affected by Water Source and Frog Source

**FIGURE 2:**
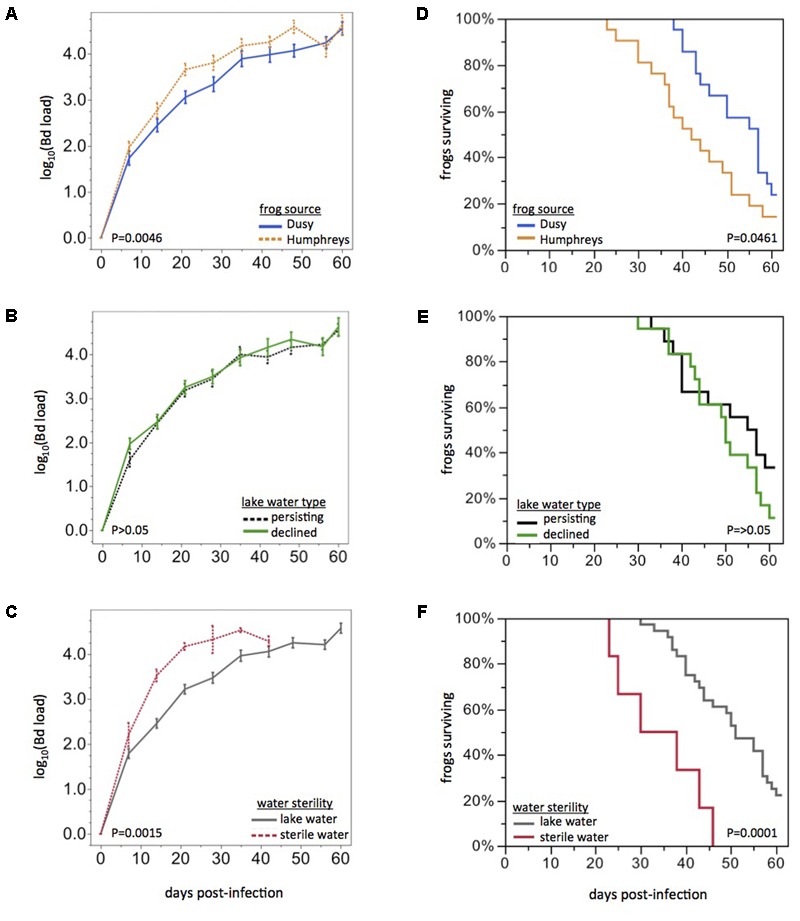
Effects of frog source and water source on disease dynamics. Bd load trajectories and survival of frogs, grouped by Frog Source (top), Lake Water Type (middle), or Water Sterility (bottom). Rates of Bd load increase and survival curves were affected by Frog Source **(A,D)** and Water Sterility **(C,F)** but not Lake Water Type **(B,E)**. Statistical tests are based on the first 21 days post-exposure only because of missing data (due to mortality) beyond that time span. All frogs eventually developed high Bd loads.

Frogs from Humphreys Basin showed more rapid rates of Bd load increase than frogs from Dusy Basin (repeated measures ANOVA: *F*_1,34_ = 7.43, *P*_Frog_ = 0.0100, *F*_3,32_ = 5.25, *P*_Frog x Time_ = 0.0046, **Figure 2A**). Water Source also affected Bd load trajectories (*F*_6,34_ = 3.18, *P*_Water Source_ = 0.0139, Wilk’s Lambda approximate *F*_18,91_ = 2.06, *P*_Water Source x Time_ = 0.0135). The significant Water Source effect was primarily driven by a difference between sterile water and lake water: if the sterile water treatment was excluded, Water Source and the Water Source x Time effects were no longer significant. The disease dynamics of field sites from which water was collected (Lake Water Type: persistent or die-off) had no effect on Bd load trajectories (*P* > 0.05, **Figure 2B**). Bd load dynamics did differ between frogs housed in sterile water compared with frogs housed in live lake water (*F*_1,34_ = 15.99, *P*_Water Sterility_ = 0.0003, *F*_3,32_ = 6.47, *P*_Water Sterility x Time_ = 0.0015, **Figure 2C**). Patterns in frog survival were consistent with Bd load trajectories: Kaplan Meier survival curves differed based on Frog Source (Chi-square: log rank test, *P* = 0.0461; Wilcoxon test, *P* = 0.0108) but not Lake Water Type (*P* > 0.05, **Figures 2D,E**). Water Sterility affected survival curves (log rank, *P* < 0.0001; Wilcoxon, *P* < 0.0001, **Figure 2F**). Notably, any differences we observed in Bd load trajectories or survival curves were only quantitative: the rate of Bd load increase or time to mortality differed slightly but all Bd-exposed frogs became infected and eventually developed Bd loads in the lethal range. Mortality of infected animals would likely have been 100% if we had not intervened with antifungal treatment.

### No Direct Evidence for Microbiome Affecting Bd Infection

Differences in skin bacterial communities prior to Bd challenge were not correlated with Bd infection intensity at 21 days PE (Mantel test: *P* > 0.05). In addition, bacterial diversity prior to Bd exposure was not correlated with downstream Bd load. Nor did individual analyses of phylotypes identify any phylotypes for which relative abundance prior to Bd infection significantly predicted downstream Bd load.

### Bacterial Communities Were Predicted by Intensity of Bd Infection

**FIGURE 3:**
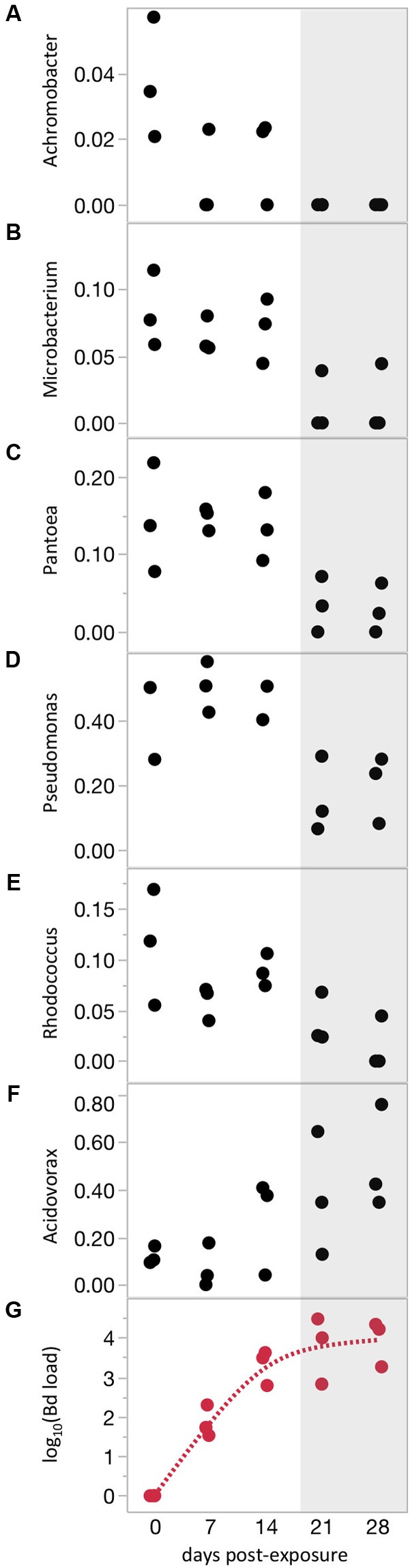
Bacterial phylotypes shift with increasing Bd severity. **(A–F)** Relative abundances of phylotypes with significant correlations with time over the course of Bd infection. **(G)** Bd load trajectory. Shaded area highlights region of highest Bd loads. All correlations shown are statistically significant (*P* < 0.05, *Q* < 0.05). Relative abundance data are arcsine(square root) transformed.

Differences in frog skin microbiomes at 21 days PE were correlated with Bd infection intensity measured on the same day (21 days PE), indicating that the intensity of Bd infection, not just Bd infection in a binary sense, influences bacterial communities (Mantel test: *P* = 0.0037, Spearman’s rank correlation coefficient = 0.15). Across all infected frogs on that same day, linear mixed models testing the effects of Bd load on relative abundance of each bacterial phylotype yielded no significant results after adjusting for multiple tests. The lack of individual phylotypes correlated with Bd load does not contradict the significant whole-community correlation with Bd load (Mantel test). Instead, it may simply indicate that the whole-community correlation with Bd load is due to small changes in many OTUs, rather than dramatic changes in a few OTUs. The lack of significant change in individual phylotypes could also be due to high intra-individual variation due to the many experimental treatments (7 Water Sources × 2 Frog Sources). Indeed, when we eliminated variation due to Frog Source and Water Source by analyzing a subset of frogs from one treatment through time, we found that relative abundances of several individual phylotypes changed as Bd loads increased over time (**Figure 3** and Supplementary Table S3). The identities of these phylotypes were highly consistent with the OTUs that responded to Bd infection when analyzed as a binary variable (infected versus uninfected, Jani and Briggs, 2014 and Supplementary Tables S2, S3). For many of these phylotypes, dramatic changes in relative abundance occurred only after Bd loads were quite high (mean above 1,000 Bd cells/swab, **Figure 3**). None of these phylotypes were correlated with time in the Bd-free frogs.

### Bacterial Diversity Was Affected by Lake Water Type

**FIGURE 4:**
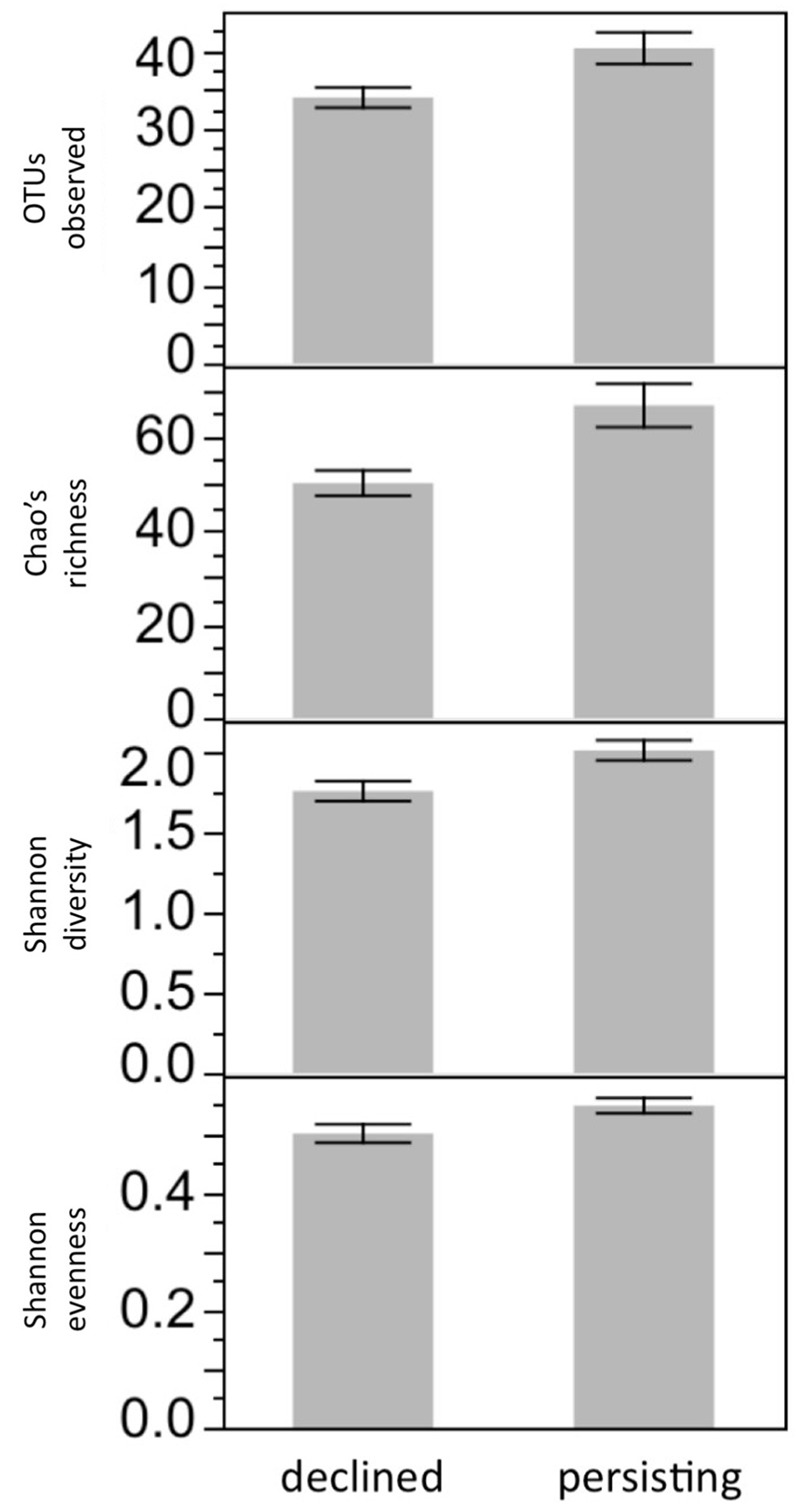
Microbiome diversity is affected by Lake Water Type. After 3 weeks of exposure to water treatments, bacterial diversity was higher on *R. sierrae* housed in water that had been collected from lakes inhabited by persisting frog populations, compared with frogs housed in water collected from lakes that had been inhabited by populations that died out due to Bd. *P* < 0.05 for all four richness or diversity metrics.

Bacterial diversity was not affected by Frog Source or Water Sterility (all *P* > 0.05). In contrast, bacterial diversity was affected by Lake Water Type: after 3 weeks in their respective lake water treatments, frogs housed in Persistent water had higher bacterial richness than frogs housed in Die-off water (*F*_1,60_ = 7.41, S_OBS_ *P* = 0.0083, *F*_1,60_ = 10.29, Chao’s richness *P* = 0.0021, *F*_1,60_ = 8.30, Shannon diversity *P* = 0.0054, *F*_1,60_ = 5.32, Shannon evenness *P* = 0.0243; **Figure 4**), seeming to mimic patterns observed in the wild (Jani et al., 2017). The difference in richness between frogs in the two types of water was due to richness declining over time in the lab more rapidly for frogs housed in die-off water than in persistent water. The decline in richness was not due to transition from the wild to captivity, as these frogs were housed in captivity for a year prior to this study. The decline might have been due to the transition from common garden tanks prior to the experiment to solitary tanks used in the experiment, if for example contact among frogs replenishes the microbiome. Another 3 weeks later, after Bd exposure, richness no longer differed between these groups. This loss of the difference in richness between frogs in Persistent and Die-off water was not explained by Bd infection: there was no significant interaction between Bd infection and Lake Water Type. Nor was bacterial diversity at 21 days PE correlated with Bd load.

### Symptoms Caused by Bd Infection

All frogs in the Bd+ treatment became infected with Bd, and Bd loads increased rapidly with time (Supplementary Figure S3a). Increases in Bd load were accompanied by weight loss: Frogs in the Bd- group appeared to gain weight over the course of the experiment, while frogs in the Bd+ group lost weight (repeated measures ANOVA with Bd treatment, Frog Source, and Water Source as explanatory variables; *P*_BdxTime_ < 0.0001, Supplementary Figure S3b). All frogs in the Bd- group survived the experiment, but the Bd+ group experienced considerable mortality (Supplementary Figure S3c). We used ANOVA models to formally test effects of Bd infection on frog appetite (number of crickets eaten per week) and skin shedding measured (based on an ordinal 3-level rating system) at 6 and 8 weeks post infection. Frog Source and Water Source were included as additional explanatory variables in the model. Bd infection led to reduced appetite (*P* < 0.0001 at 6 and 8 weeks PE, Supplementary Figure S4a) and increased skin sloughing (*P* < 0.0001 at both 6 and 8 weeks PE, Supplementary Figure S4b).

### Bacterial Communities in Stored Lake Water

Water collected from field sites maintained consistent differences due to Water Source despite storage (Pseudo-*F*_5,12_= 5.23, *P*_Water Source_ = 0.0001) and was consistent through time (Supplementary Figure S5). Richness and composition of bacterial communities in water did not differ by Lake Water Type (persistent or die-off, *P* > 0.05 for all tests). However, one measure of richness was marginally higher in persistent water (*P*_SOBS_ = 0.0860), and lack of significance in other metrics is not conclusive because power to detect differences was low (Supplementary Figure S5).

## Discussion

### Summary

Wild *R. sierrae* populations exhibit distinct disease dynamics, either persisting or experiencing catastrophic declines in response to Bd infection. These differences in disease dynamics in wild populations coincide with differences in the composition of skin-associated bacterial communities (Jani et al., 2017). To understand the biological meaning and conservation relevance of these patterns, we need to tease apart cause and effect in interactions between Bd and the microbiome and improve understanding of what shapes the microbiome in the first place. With the current experiment, we aimed to identify factors that shape the *R. sierrae* skin microbiome, and to clarify causal Bd-bacteria relationships. Our results demonstrate that both host background and the aquatic environment affect the skin microbiome. We found that innate (i.e., inherent) differences between conspecific populations led to differences in the skin microbiome. We also found that lake water to which frogs are exposed can shape differences in the skin microbiome, even in the absence of additional environmental factors such as sediment, vegetation, or contact with other amphibians. Host background also predicted variation in Bd load increase and time to mortality. However, we found no direct evidence that the microbiome confers resistance to Bd, at least in this laboratory setting. Thus, although our experimentally altered microbiomes were correlated with downstream Bd dynamics, we cannot conclude that the microbiome was causally linked to Bd dynamics.

### Frog Population Affects Skin Microbiome and Bd Infection Trajectory

**FIGURE 5:**
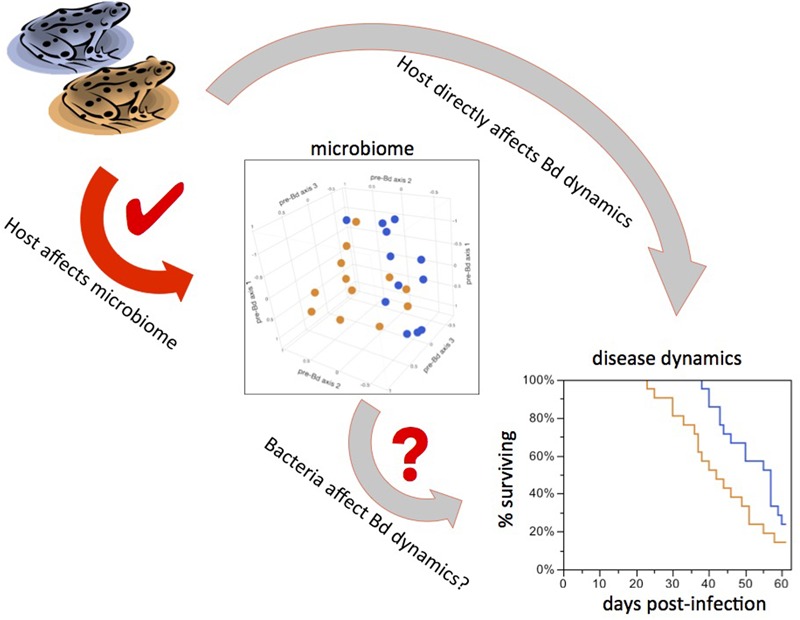
Teasing apart cause from correlation. Possible causal links between experimental treatments and Bd infection outcomes. Frog sources led to different bacterial communities and differences in downstream Bd dynamics (red arrow). This could mean that bacteria mediate disease dynamics (short gray arrow). It is also possible that the host influences both the microbiome and disease dynamics independently of each other (long gray arrow), such that the variation in bacteria is not the driver of differences in Bd dynamics.

Previous work showed differences between microbiomes of amphibian species sharing a common lake environment (McKenzie et al., 2012; Kueneman et al., 2014; Walke et al., 2014). Here, we show that within-species variation in the microbiome is at least partly controlled by innate (most likely genetic) differences between host populations. Furthermore, the effect of Frog Source was significant both in the absence of Bd and after Bd infection, indicating that the disturbance to the microbiome caused by Bd infection does not completely override host effects on the microbiome. We also showed that the rate of increase in Bd loads in the experiment differed between frogs from the two populations, demonstrating that host-controlled microbiome variation is associated with host response to Bd. Thus there is potential for a cascade of effects in which host background influences the microbiome, which in turn affects infection dynamics. However, it is also possible that frog population background or age affects disease dynamics through unknown mechanisms independent of the skin microbiome (**Figure 5**). Isolating the effect of the microbiome is extremely difficult – particularly in cases such as this, when we are interested in subtle differences representative of natural variation – and presents an important challenge for future research.

Using a controlled, factorial experiment enables us to isolate the role of the host in shaping the microbiome. We included frogs from two different source populations to test how different genetic backgrounds affect the microbiome. However, in this study, the frogs from the two populations were of slightly different ages, therefore we cannot completely rule out the possibility that age played a role in microbiome differences. However, previous studies of *R. sierrae* and its close relative *Rana cascadae* found no difference in the microbiomes of juveniles compared with adults (Jani and Briggs, 2014; Kueneman et al., 2014). In the current study the age variation was relatively small, with all frogs being juveniles, therefore we do not expect the age difference to be the cause of the observed differences in microbiomes between the two population sources. We conclude that in this study genetic differences are a more likely driver of the microbiome differences between the two frog populations.

In this study, initial rates of Bd load increase varied, but no frogs fully resisted or cleared Bd infection, and all frogs challenged with Bd eventually developed Bd loads in the lethal range. Thus, none of the experimental treatments conferred true resistance to infection or disease. While we had hoped that exposing frogs to aquatic bacteria from persistent sites would confer some resistance to Bd, it is not surprising that neither of the frog populations exhibited inherent resistance to disease given that wild populations in both Dusy Basin and Humphreys Basin have collapsed due to Bd. However, differences in rates of Bd load increase may have practical importance even in the absence of complete disease resistance. In recent years, researchers have attempted interventions using antifungal drugs or probiotics to curb the devastating effects of Bd on *R. sierrae* populations (Knapp et al., unpublished). These efforts depend critically on researchers and managers having sufficient time to mobilize intervention efforts before frogs become too heavily infected with Bd. Thus, even without complete resistance, variation in the rate of increase in Bd infection intensity can have practical conservation implications. In the current study we found frogs from Dusy Basin and Humphreys Basin populations harbor different bacterial communities, and also exhibit subtly different rates of increase in Bd loads. These results highlight the importance of further research to understand host population level differences, both in terms of host genetics and associated symbiotic microbes. The populations in the current study were closely related, both belonging to the same *R. sierrae* clade (Vredenburg et al., 2007) and both suffering the same fate due to Bd in the wild. We speculate that laboratory comparisons of more distantly related populations (e.g., persisting northern populations compared with declining southern populations) would reveal more dramatic differences in microbiome composition and disease resistance.

### Lake Water Type Affects Skin Microbiome But Not Bd Infection Trajectory

A key finding of our study is that housing frogs in water from different lakes was sufficient to mimic some microbiome patterns in the field: namely, differences between persistent and die-off *R. sierrae* populations. In the field, *R. sierrae* populations that persist with Bd harbor different bacterial communities than populations that died out due to Bd. Similarly, in the current laboratory experiment, frogs developed different bacterial communities depending on whether they were housed in water collected from field sites inhabited by persistent or die-off populations. This result is remarkable given the limitations of our mesocosms: the mesocosms consisted simply of tanks with water from different lakes. No vegetation or sediment from field sites was added. Despite their limitations, our mesocosms induced differences in the bacterial communities on *R. sierrae* skin that were consistent with field patterns. Notably, all frogs in this experiment were from die-off populations, which is important because frog source and water source are not confounded and we can conclude that the differences observed between different water treatments are indeed due to those water treatments. In contrast, in field surveys we cannot conclude whether differences in the microbiomes of persistent and die-off populations is due to the environment or frog genetic background, or both. Notably, in field surveys the difference between microflora of persistent and die-off populations is more dramatic than in the current experiment, indicating that not all of the variation observed in the field is captured by our experiment. We think it is most likely that, in addition to the aquatic environment, host genetic background contributes to differences between die-off and persistent populations in the field, although additional environmental variables not tested in our experiment (such as lake sediment) likely also play a role. Our results demonstrate that water is an important environmental driver of variation in the microbiome, and differences in water sources alone can explain some of the variation in microbiomes between persistent and die-off *R. sierrae* populations in the field.

Although frogs housed in water from persistent and die-off water developed different microbiomes, this did not translate to any difference in the rate of Bd load increase. Thus, to the extent that our mesocosms represent environmental variation, we found no evidence supporting the hypothesis that environmentally mediated differences in the microbiome determine *R. sierrae* populations persistence or decline due to chytridiomycosis. We therefore speculate that host genetic differences may be more important than environmental drivers in determining *R. sierrae* population response to Bd infection.

### Water Sterility Affects the Microbiome and Downstream Bd Dynamics

This experiment also demonstrated differences in the overall composition of microbiomes of frogs housed in sterile compared with non-sterile aquatic environments. These results are consistent with previous studies (Loudon et al., 2013; Knutie et al., 2017). In addition to affecting the *R. sierrae* microbiome, the experimental aquatic environment affected Bd infection trajectories. Increases in Bd loads were more rapid in frogs housed in sterile water than frogs housed in live lake water. As with differences in frog genetic background, the tentative conclusion of a cascade of effects from aquatic environment to skin microbiome to disease dynamics is only one possible interpretation of the data, and it is important to consider alternative explanations. For example, in addition to harboring different bacterial communities, water sources may vary in water chemistry, which may affect the *R. sierrae* microbiome. Another possible explanation for why Bd dynamics differed between live and sterile water treatments is that Bd survival in the aquatic habitat is directly affected by Water Sterility. Re-infection of a frog by zoospores released from its own skin to the water probably contributed to Bd infection dynamics in this experiment. If water bacterial communities or chemistry affect Bd zoospore survival, this could lead to differences in the density of infective zoospores in the aquatic environment, affecting the rate of Bd load increase on frogs. In the latter case, Water Sterility affects the frog microbiome and also independently affects disease dynamics, without there necessarily being a causal link between the frog microbiome and disease dynamics. Studies have found that grazing by aquatic crustaceans affects Bd zoospore densities (Hamilton et al., 2012; Searle et al., 2013; Kagami et al., 2014). In the current study, we filtered all macro-organisms from lake water, but bacteria present in the water may interact with Bd, and presence of organic matter, albeit minimal in these oligotrophic lakes, may provide resources for Bd growth or survival. Thus we cannot conclude with certainty that the differences in disease trajectories between live and sterile water treatments were caused by a cascade of effects from aquatic bacteria to frog microbiome to disease dynamics. Additional studies examining growth and survival of Bd in different aquatic environments may help clarify the effects of aquatic bacteria on Bd.

### Does Microbiome Diversity Increase Disease Resistance?

The source of lake water in this experiment affected the richness of the *R. sierrae* skin microbiome. Notably, these effects mimicked patterns observed in the field: frogs housed in water from persistent (northern) populations harbored higher bacterial richness than frogs housed in water from die-off (southern) populations (Jani et al., 2017). However, in the current experiment the higher richness on frogs in Persistent water did not prevent or lessen Bd infection when frogs were subsequently exposed to Bd. In contrast, previous experimental work showed that greater bacterial diversity reduces Bd growth *in vitro* (Piovia-Scott et al., 2017). In addition, field observations showed that northern (persistent) *R. sierrae* populations have greater diversity than southern populations, which generally suffer die-offs due to Bd infection. Based on these previous findings we hypothesized that microbiome richness may play a protective role in *R. sierrae*. Our results in the current study fail to support that conclusion. However, there are at least four plausible explanations for our results. The results may mean that richness is in fact not protective, and that the richness – persistence relationship in the field is not causal. A second possibility is that diversity does increase disease resistance in the wild, but that the effect is not due to diversity *per se* but rather due to the increased likelihood of key taxa being present. If those key taxa are uncommon in the laboratory environment, then richness may not confer protection in the lab, even if it does in the field. A third hypothesis is that richness needs to reach a minimum threshold to provide protection. Richness in the field was much higher than in the current laboratory study, so it is possible that even the highest richness observed in the lab was insufficient to confer protection. (Mean Chao’s richness in persistent water was 66 in current experiment and ∼250 in field, see Figure 4c in Jani et al., 2017.) Finally it is possible that the magnitude of the difference in richness in the current experiment was not great enough to result in differences in disease resistance between the experimental groups. In the field, frogs in northern sites had about twice the richness of southern frogs, while frogs in persistent water in the current study had only about a 30% increase in richness compared with frogs in die-off water. Thus, the current experiment does not support a protective role for bacterial diversity, but does not rule it out either. We have observed that microbiome diversity patterns are less consistent than patterns based on composition, suggesting that the identity of bacteria is important, rather than simply the number of species and evenness of their abundances. Repeating studies that examine microbiome diversity will be important for forming firm conclusions.

### Bd Disturbance Depends on Bd Load

Analyses of time series data over the course of increasing Bd infection identified several phylotypes that appeared to be affected by increasing Bd loads, suggesting that certain bacteria respond to the severity of Bd infection, not simply to the presence or absence of infection. Notably, for several phylotypes, the effects of Bd was most pronounced after Bd loads reached high levels (**Figure 3**). Previous studies have found effects of Bd infection on the microbiome (Jani and Briggs, 2014; Longo et al., 2015; Jani et al., 2017), while another case found no effect (Becker et al., 2015). The latter study tested for effects of Bd on the microbiome early in infection when loads were low. Our current finding that the effect of Bd on bacteria depends on Bd load suggests that discrepancies between previous studies could be in part due to the fact that studies examine Bd effects at different Bd loads.

### Strengths and Limitations of Laboratory Experiments

Laboratory experiments cannot reproduce natural settings. Laboratory storage of lake water alters aquatic microbial communities, and captivity alters the *R. sierrae* skin microbiome. However, microbial community composition was more strongly predicted by sample type (*R. sierrae* versus water) than by location (lab versus field). *R. sierrae* microbiomes from the field and lab were more similar to each other than to aquatic microbial communities (Supplementary Results and Supplementary Figure S1 in Jani and Briggs, 2014). In contrast, in other study systems, the shift between captive and wild microbiomes can be more dramatic. For example, microbiomes of terrestrial salamanders in the wild were more similar to field soil bacterial communities than to the microbiomes of the same animals in captivity (Loudon et al., 2013). In general, laboratory studies cannot be assumed to exactly reproduce field phenomena. The strengths of laboratory experiments lie in the ability to randomize and assign treatments, thereby clarifying cause and effect and disentangling factors that often co-vary in the field. Field studies have shown that persistent *R. sierrae* populations have different microbiomes from die-off populations, but wild populations differ in both genetic background and environmental conditions (Jani et al., 2017). The current experiment complements prior field results by separating host and environmental drivers of microbiome variation and directly testing links to disease outcomes.

### Teasing Apart Cause and Effect in Correlations Between Bd and the Microbiome

Surveys of wild populations of *R. sierrae* and its close relative *Rana muscosa* have found correlations between population response to Bd (persistence or decline) and skin-associated bacterial communities (Woodhams et al., 2007; Jani et al., 2017). Bd has also been shown to disturb the microbiome (Jani and Briggs, 2014). It is therefore impossible to definitively determine from field surveys of infected populations whether correlations between bacteria and Bd load are due to variation in protective effects of bacteria or Bd-induced disturbance of the microbiome. In the current study, we used a controlled experiment to tease apart cause and effect. We found that the severity of Bd infection is significantly correlated with overall composition of the microbiome after Bd infection, but not before Bd infection. These results indicate that in this experiment the effect of Bd infection on the microbiome was stronger than the effect of the microbiome on Bd infection. However in this study variation in response to disease was limited: despite variation in rates of disease progression, all frogs in this study were susceptible to chytridiomycosis. Similar experiments comparing populations or species that show greater distinction in resistance to Bd would provide valuable additional insight to the role of the amphibian microbiome in disease resistance.

## Ethics Statement

This study was carried out in accordance with the recommendations of Institutional Animal Care and Use Committee of the University of California, Santa Barbara (UCSB IACUC). All animal procedures were approved by the UCSB IACUC.

## Author Contributions

AJ and CB designed the study. AJ performed the experiments and analyses and wrote the paper with revisions from CB.

## Conflict of Interest Statement

The authors declare that the research was conducted in the absence of any commercial or financial relationships that could be construed as a potential conflict of interest.
