# Supplementary material for: Host and Aquatic Environment Shape the Amphibian Skin Microbiome but Effects on Downstream Resistance to the Pathogen Batrachochytrium dendrobatidis Are Variable
Source: Front Microbiol. 2018 Mar 21;9:487. doi: 10.3389/fmicb.2018.00487 (PMC5871691; doi:10.3389/fmicb.2018.00487)
Supplement: Supplementary file 1 [file Table_1.docx]

**Table S1.** Comparison of results of analyses based on phylotypes or 95% identity OTUs. Classification is the summary taxonomy of each phylotype or OTU. “increase” or “decrease” indicates response to Bd infection as a binary variable. “Phylotypes” column shows effect of Bd infection when bacteria are grouped by phylotype (generally genus). “OTUs” column shows effect of Bd infection when bacteria are defined by 95% sequence identity, as in Jani and Briggs 2014. NS – not statistically significant.

| **Classification** | **Phylotypes** | **OTUs** |
| --- | --- | --- |
| Actinobacteria-Microbacteriaceae-*Microbacterium* | decrease | decrease |
| Actinobacteria-Nocardiaceae-*Rhodococcus* | decrease | decrease |
| Actinobacteria-Sanguibacteraceae-*Sanguibacter* | decrease | decrease |
| Alphaproteobacteria-Brucellaceae-*Ochrobactrum* | decrease | decrease |
| Betaproteobacteria-Comamonadaceae-*Acidovorax* | increase | increase |
| Betaproteobacteria-Comamonadaceae-*Curvibacter* | increase | increase |
| Betaproteobacteria-Comamonadaceae-*Pseudorhodoferax* | increase | NS |
| Betaproteobacteria-Comamonadaceae-*Rhodoferax* | increase | increase |
| Betaproteobacteria-Methylophilaceae-*Methylotenera* | decrease | decrease |
| Betaproteobacteria-Neisseriaceae-*Aquitalea* | decrease | decrease |
| Betaproteobacteria-Oxalobacteraceae-*Duganella* | decrease | NS |
| Betaproteobacteria-Oxalobacteraceae-*Janthinobacterium* | increase | increase |
| Betaproteobacteria-Oxalobacteraceae-*Undibacterium* | increase | increase |
| Gammaproteobacteria-Enterobacteriaceae-*Enterobacter* | decrease | NS |
| Gammaproteobacteria-Enterobacteriaceae-*Pantoea* | decrease | decrease |
| Gammaproteobacteria-Pseudomonadaceae-*Pseudomonas* | decrease | decrease |
| Gammaproteobacteria-Xanthomonadaceae-*Stenotrophomonas* | decrease | decrease |
