# Supplementary material for: Host and Aquatic Environment Shape the Amphibian Skin Microbiome but Effects on Downstream Resistance to the Pathogen Batrachochytrium dendrobatidis Are Variable
Source: Front Microbiol. 2018 Mar 21;9:487. doi: 10.3389/fmicb.2018.00487 (PMC5871691; doi:10.3389/fmicb.2018.00487)
Supplement: Supplementary file 2 [file Table_2.docx]

**Table S2.** Phylotypes affected by Frog or Water treatments in the experiment. Each cell specifies the treatment in which the relative abundance of a given phylotype was higher. ** Statistically significant (P<0.05, Q<0.05), * Marginally statistically significant (P<0.05, 0.05<Q<0.10).

| **Phylotype** | **Frog Source** | **Lake Water Type** | **Water Sterility** |
| --- | --- | --- | --- |
| Actinobacteria-Nocardiaceae-*Rhodococcus* |  | **persistent |  |
| Actinobacteria-Sporichthyaceae-hgcI_clade |  |  | **lake water |
| Alphaproteobacteria-Bradyrhizobiaceae-*Bosea* |  | **die-off | **sterile water |
| Alphaproteobacteria-Bradyrhizobiaceae-*Rhodopseudomonas* |  | *die-off |  |
| Alphaproteobacteria-Caulobacteraceae-*Phenylobacterium* |  | *persistent | *sterile water |
| Alphaproteobacteria-Phyllobacteriaceae-*Hoeflea* |  | **die-off |  |
| Alphaproteobacteria-Rhizobiaceae-*Agrobacterium* |  |  | *sterile water |
| Alphaproteobacteria-Rhizobiaceae-*Rhizobium* | *Humphreys |  |  |
| Alphaproteobacteria-Rhizobiaceae-*Shinella* |  |  | *sterile water |
| Betaproteobacteria-Burkholderiaceae-*Polynucleobacter* |  |  | **sterile water |
| Betaproteobacteria-Comamonadaceae-*Acidovorax* | **Humphreys | **persistent | **sterile water |
| Betaproteobacteria-Comamonadaceae-*Albidiferax* |  | **persistent |  |
| Betaproteobacteria-Comamonadaceae-*Brachymonas* |  | *die-off |  |
| Betaproteobacteria-Comamonadaceae-*Chlorochromatium* |  | **persistent |  |
| Betaproteobacteria-Comamonadaceae-*Comamonas* |  | **persistent |  |
| Betaproteobacteria-Comamonadaceae-*Delftia* |  |  | *sterile water |
| Betaproteobacteria-Comamonadaceae-*Limnohabitans* |  | *persistent |  |
| Betaproteobacteria-Comamonadaceae-*Paucibacter* |  |  | *lake water |
| Betaproteobacteria-Methylophilaceae-*Methylotenera* |  | **persistent |  |
| Betaproteobacteria-Methylophilaceae-PRD01a011B |  | **persistent |  |
| Betaproteobacteria-Neisseriaceae-*Vogesella* |  | **persistent |  |
| Betaproteobacteria-Oxalobacteraceae-*Duganella* |  | *die-off |  |
| Betaproteobacteria-Oxalobacteraceae-*Herbaspirillum* |  | **die-off |  |
| Betaproteobacteria-Rhodocyclaceae-*Dechloromonas* |  | *die-off |  |
| Betaproteobacteria-unclassified-unclassified | **Humphreys |  |  |
| Cytophagia-Cytophagaceae-*Arcicella* |  |  | **sterile water |
| Flavobacteria-Cryomorphaceae-*Fluviicola* |  |  | **sterile water |
| Flavobacteria-Flavobacteriaceae-*Chryseobacterium* |  |  | *lake water |
| Flavobacteria-Flavobacteriaceae-*Flavobacterium* |  |  | *lake water |
| Gammaproteobacteria-Enterobacteriaceae-*Citrobacter* |  | *persistent |  |
| Gammaproteobacteria-Enterobacteriaceae-Escherichia-*Shigella* |  |  | **lake water |
| Gammaproteobacteria-Enterobacteriaceae-*Klebsiella* |  |  | **sterile water |
| Gammaproteobacteria-Enterobacteriaceae-*Yokenella* |  | **persistent |  |
| Gammaproteobacteria-Moraxellaceae-*Acinetobacter* |  | **persistent | **sterile water |
| Gammaproteobacteria-Pseudomonadaceae-*Pseudomonas* |  | **persistent |  |
| Gammaproteobacteria-Xanthomonadaceae-*Pseudoxanthomonas* |  |  | **sterile water |
| Sphingobacteriia-env.OPS_17-unclassified |  | **die-off |  |
| Sphingobacteriia-Sphingobacteriaceae-*Sphingobacterium* |  |  | **lake water |
| Verrucomicrobiae-Verrucomicrobiaceae-*Luteolibacter* | **Humphreys |  |  |
