# Supplementary material for: Host and Aquatic Environment Shape the Amphibian Skin Microbiome but Effects on Downstream Resistance to the Pathogen Batrachochytrium dendrobatidis Are Variable
Source: Front Microbiol. 2018 Mar 21;9:487. doi: 10.3389/fmicb.2018.00487 (PMC5871691; doi:10.3389/fmicb.2018.00487)
Supplement: Supplementary file 3 [file Table_3.docx]

**Table S3.** Phylotypes that changed over time as Bd loads increased. For this table analyses include data from 3 frogs, which were all Bd-exposed and from the same Frog Source and Water Source, therefore correlations with time or Bd are not confounded by differences other experimental variables. **ŧ** denotes phylotypes that were also affected by Bd infection as a binary variable (infected versus uninfected, Table S1 and Jani and Briggs 2014). ** Statistically significant (P<0.05, Q<0.05), * Marginally statisically significant (P<0.05, 0.05<Q<0.10).

| **Phylotype** | **Change through time** |
| --- | --- |
| Actinobacteria-Microbacteriaceae-*Microbacterium* | ŧ** decrease |
| Actinobacteria-Nocardiaceae-*Rhodococcus* | ŧ** decrease |
| Actinobacteria-Sanguibacteraceae-*Sanguibacter* | ŧ* decrease |
| Betaproteobacteria-Alcaligenaceae-*Achromobacter* | ** decrease |
| Betaproteobacteria-Comamonadaceae-*Acidovorax* | ŧ** increase |
| Betaproteobacteria-Neisseriaceae-*Aquitalea* | ŧ* decrease |
| Cytophagia-Cytophagaceae-*Flectobacillus* | * increase |
| Gammaproteobacteria-Enterobacteriaceae-*Pantoea* | ŧ** decrease |
| Gammaproteobacteria-Pseudomonadaceae-*Pseudomonas* | ŧ** decrease |
