# Supplementary material for: Host and Aquatic Environment Shape the Amphibian Skin Microbiome but Effects on Downstream Resistance to the Pathogen Batrachochytrium dendrobatidis Are Variable
Source: Front Microbiol. 2018 Mar 21;9:487. doi: 10.3389/fmicb.2018.00487 (PMC5871691; doi:10.3389/fmicb.2018.00487)

SUPPLEMENTARY FIGURES

Figure S1. Experimental timeline (top) and description of factors manipulated in the experiment (tables, bottom). Asterisks indicate days on which swabs were collected. Time series analysis arrow indicates period over which weekly microbiome samples were analyzed for the subset of 6 frogs (3 Bd+, 3Bd-, all of the same Frog Source and Water Source treatment). "All swabs sequenced" indicates that 16S sequence data were analyzed for all frog (N=84).

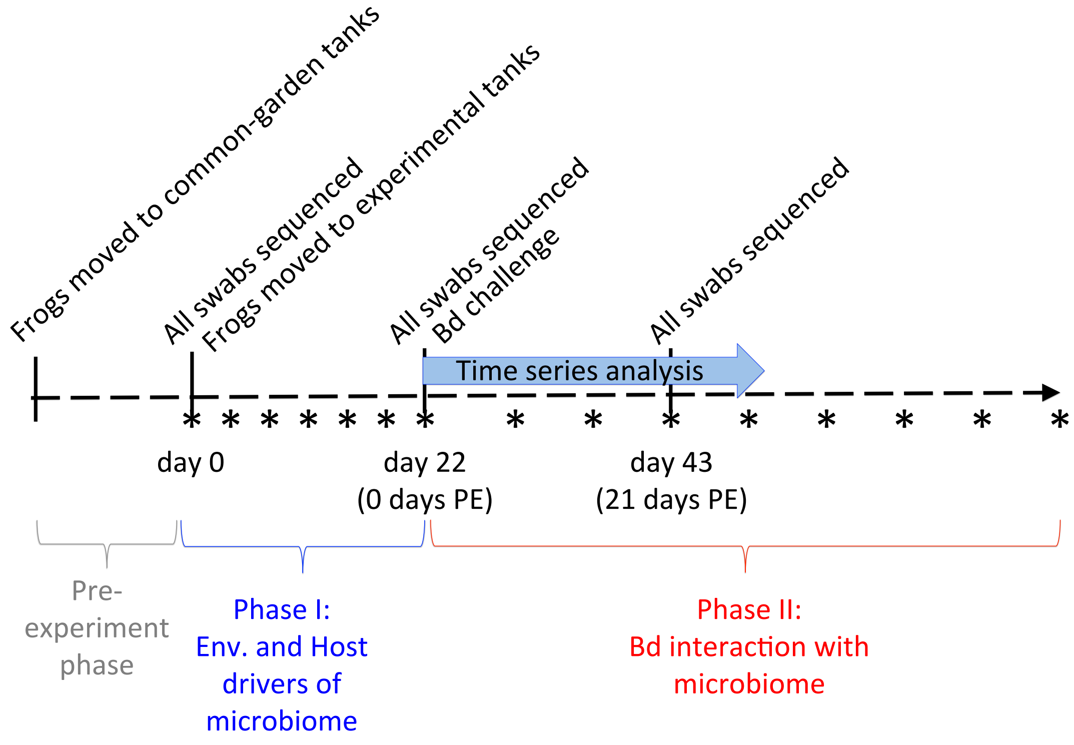

Experimental Factors:

factor	levels	description
Frog Source	2	Population from which frogs originated.
Water Source	7	Source of water in which frogs were housed. These included 6 natural lake sources and 1 sterile water treatment.
Lake Water Type	2	Persistent or Die-off: Refers to the disease dynamics of populations inhabiting lakes from which lake water was collected to house frogs in this experiment. Six of the 7 Water Sources are nested in Lake Water Type.
Water Sterility	2	Sterile bottled water or Live lake water. The former is represented by 1 Water Source while the later encompasses the 6 Water Sources that were collected from lakes.
Bd Exposure	2	Bd-exposed (infected) or unexposed (uninfected).

Experiment fully crossed 2 Frog Sources, 7 Water Sources, and 2 Bd treatments. N=6 frogs/treatment before Bd exposure, N=3 frogs/treatment after Bd exposure. Total N=84. Water Source was nested in Lake Water Type and Water Sterility.

Diagram showing nested structure of water-related variables:

<table>
<tr><th>VARIABLE:</th><th>WATER STERILITY (2 LEVELS)</th><th>LAKE WATER TYPE (6 LEVELS)</th><th>WATER SOURCE (7 LEVELS)</th></tr>
<tr><td rowspan="7">levels:</td><td>Sterile</td><td>N/A</td><td>sterile lab water</td></tr>
<tr><td rowspan="6">Live</td><td rowspan="3">water from persistent population sites</td><td>water from lake 1</td></tr>
<tr><td>water from lake 2</td></tr>
<tr><td>water from lake 3</td></tr>
<tr><td rowspan="3">water from die-off population sites</td><td>water from lake 4</td></tr>
<tr><td>water from lake 5</td></tr>
<tr><td>water from lake 6</td></tr>
</table>

Figure S2. Skin bacterial communities differed between frogs housed in sterile water compared with frogs housed in live lake water after three weeks exposure to experimental water treatments (a). Differences persisted after Bd infection (b). Marker color indicates water treatment (sterile or lake water). NMDS stress: (a) 0.09; (b) 0.16.

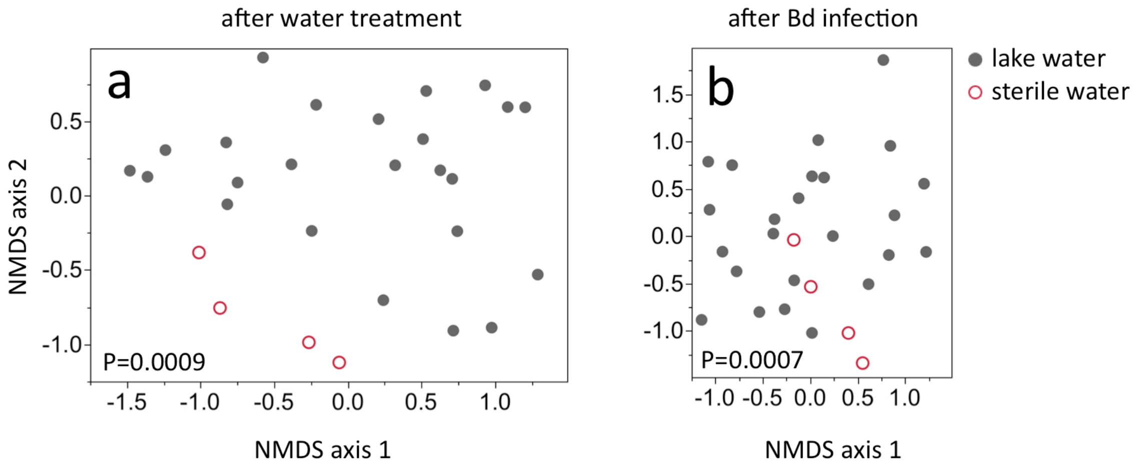

Figure S3. (a) Bd infection trajectories averaged across all Bd-exposed frogs compared with Bd-free control group. (b) Weight loss caused by Bd infection. (c) Survival curve for all Bd-exposed frogs compared with Bd-free control group.

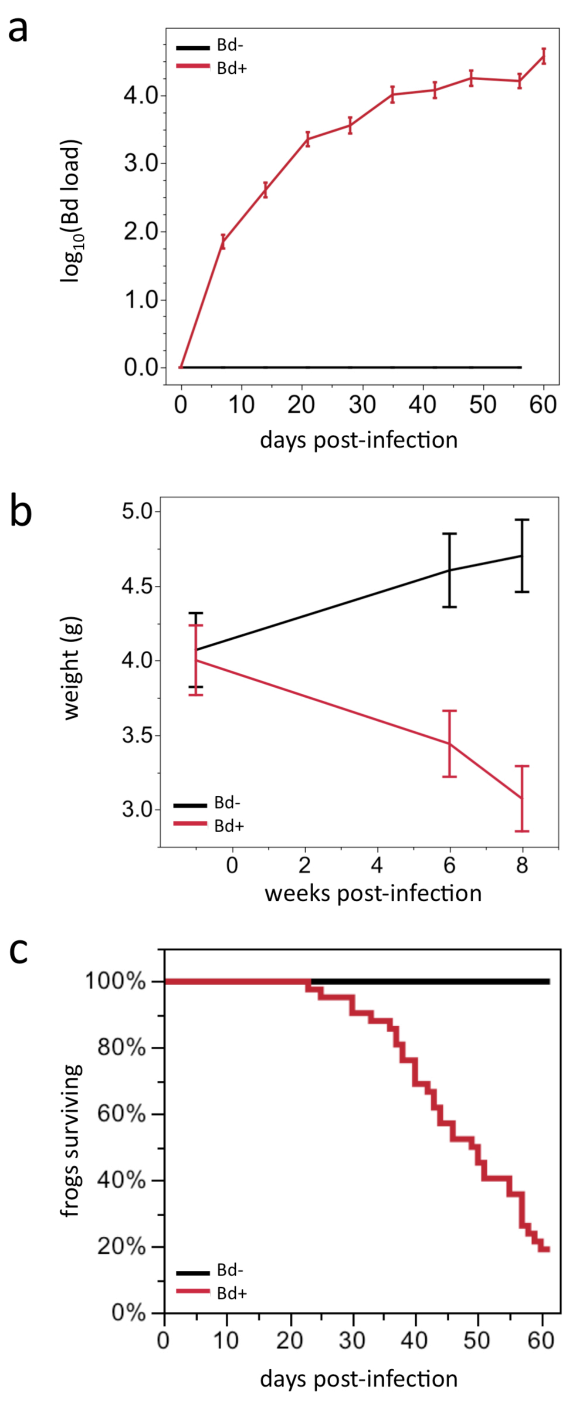

Figure S4. Infected frogs ate fewer crickets (a) and shed more skin (b) than uninfected frogs. (a) shows number of crickets eaten per week. (b) is based on a 1-to-3 qualitative visual rating of the amount of shed skin in frog tanks.

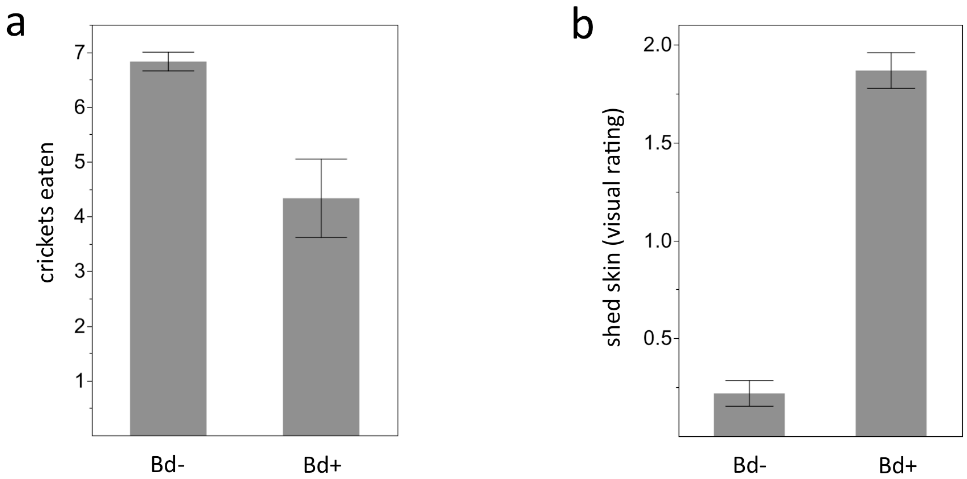

Figure S5. Bacterial communities in stored lake water. (a) Bacterial diversity in water from persistent and die-off field sites, when water is stored in the lab. Model: Y ~ LakeWaterType + DayOfExperiment. P_{Day} >0.05 for all diversity metrics. $P_{LakeWaterType}$=.0860 for SOBS, $P_{LakeWaterType}$ >0.05 for all other metrics. (b) NMDS ordination of bacterial communities. Bacterial community composition differed by Water Source but not by Day of experiment, indicating that water maintained differences due to field source and these were robust to effects of storage. PERMANOVA $P_{LakeWaterType}$>0.05, P_{Day}>0.05, $P_{WaterSource}$=0.0001. Models: Y ~ LakeWaterType + Day. Y ~ WaterSource. (Day could not be included in test of effect of water source because this would leave N=1 per WaterSource-Day combination.) Water collected from six lakes for this experiment was stored at 4°C. Water was sampled from storage tanks (without frogs). Samples collected on days 1, 20, and 34 of the experiment were sequenced to characterize the bacterial communities, as described in Methods. Note: Sample sizes for statistical tests are low (only 6 collection lakes). Ordination stress: 0.11.

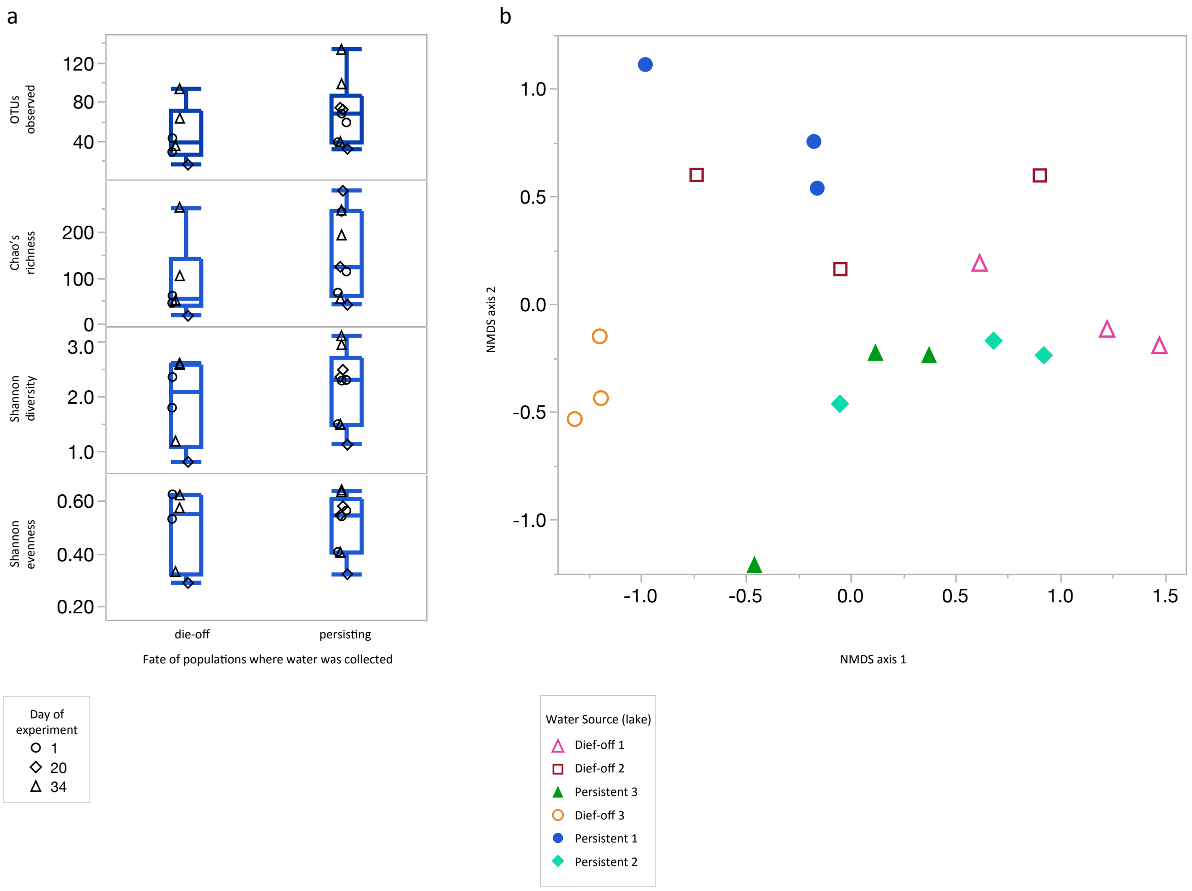

Supplement: Supplementary file 4 [file Presentation_1.PDF]
